# Role of the *Trypanosoma brucei* natural cysteine peptidase inhibitor ICP in differentiation and virulence

**DOI:** 10.1111/j.1365-2958.2007.05970.x

**Published:** 2007-10-18

**Authors:** Camila C Santos, Graham H Coombs, Ana Paula C A Lima, Jeremy C Mottram

**Affiliations:** 1 Instituto de Biofisica Carlos Chagas Filho, Universidade Federal do Rio de Janeiro, Bloco G, C.C.S., Cidade UniversitariaRio de Janeiro, RJ, 21949-900, Brazil; 2 Wellcome Centre for Molecular Parasitology, Glasgow Biomedical Research Centre, University of Glasgow120 University Place, Glasgow G12 8TA, UK.

## Abstract

ICP is a chagasin-family natural tight binding inhibitor of Clan CA, family C1 cysteine peptidases (CPs). We investigated the role of ICP in *Trypanosoma brucei* by generating bloodstream form *ICP*-deficient mutants (Δ*icp*). A threefold increase in CP activity was detected in lysates of Δ*icp*, which was restored to the levels in wild type parasites by re-expression of the gene in the null mutant. Δ*icp* displayed slower growth in culture and increased resistance to a trypanocidal synthetic CP inhibitor. More efficient exchange of the variant surface glycoprotein (VSG) to procyclin during differentiation from bloodstream to procyclic form was observed in Δ*icp*, a phenotype that was reversed in the presence of synthetic CP inhibitors. Furthermore, we showed that degradation of anti-VSG IgG is abolished when parasites are pretreated with synthetic CP inhibitors, and that parasites lacking *ICP* degrade IgG more efficiently than wild type. In addition, Δ*icp* reached higher parasitemia than wild type parasites in infected mice, suggesting that *ICP* modulates parasite infectivity. Taken together, these data suggest that CPs of *T. brucei* bloodstream form play a role in surface coat exchange during differentiation, in the degradation of internalized IgG and in parasite infectivity, and that their function is regulated by ICP.

## Introduction

Clan CA, family C1 cysteine peptidases (CPs) are considered crucial for the growth, differentiation and survival of several pathogenic protozoa (for a review, see Sajid and McKerrow, 2002). In *Trypanosoma brucei* species, the pathogenic kinetoplastid protozoa responsible for human and veterinary trypanosomiasis in sub-Saharan Africa, the major CP has primary sequence and biochemical characteristics that are broadly similar to those of mammalian cathepsin L (Lonsdale-Eccles and Grab, 1987; Troeberg *et al*., 1999; Caffrey *et al*., 2001), and is encoded by a tandem array of 11 nearly identical gene copies (Berriman *et al*., 2005). The enzymes in *T. b. rhodesiense* and *T. b. brucei* are termed rhodesain and brucipain (or trypanopain) respectively (Lonsdale-Eccles and Grab, 1987; Caffrey *et al*., 2001). It has been demonstrated that small-molecule inhibitors of CPs kill *T. b. brucei* in culture as well as in experimentally infected animals (Scory *et al*., 1999; Troeberg *et al*., 1999). Importantly, killing of the parasites was correlated with inhibition of brucipain, suggesting that this peptidase plays a crucial role in the biology of the parasite (Troeberg *et al*., 1999).

Peptidase activity can be regulated at several levels, extending from gene expression to the synthesis of inhibiting proteins. In mammals and plants, CPs are regulated by members of the cystatin family (Abrahamson *et al.*, 2003), which are absent from kinetoplastid protozoa (Ivens *et al*., 2005). A search for endogenous inhibitors of the parasites CPs resulted in the discovery of a family of inhibitors distinct from cystatins and other groups of peptidase inhibitors, which were named the chagasin family (or Inhibitors of Cysteine Peptidases, ICP) (Monteiro *et al*., 2001; Sanderson *et al*., 2003). Chagasin was initially isolated from *T. cruzi* and is a potent tight-binding inhibitor of Clan CA, family C1 CPs (Monteiro *et al*., 2001). Chagasin homologues were subsequently identified in other protozoa and in bacteria, and these genes were proven to encode functional CP inhibitors (Rigden *et al.* 2002; Sanderson *et al*., 2003; Riekenberg *et al*., 2005; Pandey *et al*., 2006). Structure determination of *Leishmania mexicana* ICP and chagasin revealed that they adopt a type of immunoglobulin (Ig)-like fold not previously reported in lower eukaryotes (Salmon *et al*., 2006; Smith *et al*., 2006; Figueiredo da Silva *et al*., 2007). It was demonstrated that, in *T. cruzi*, chagasin forms tight binding complexes with the major CP of the parasite, cruzipain (Santos *et al*., 2005). A fourfold increase in inhibitor expression in transgenic parasites led to a marked reduction in CP activity, resulting in reduced differentiation from non-infective epimastigotes to the infective trypomastigote form, increased resistance to the deleterious effect of a synthetic CP inhibitor, and diminished infectivity of tissue culture trypomastigotes *in vitro*; these data suggested that chagasin controls endogenous CP activity (Santos *et al*., 2005). In contrast, deletion of the *L. mexicana* ICP resulted in reduced infectivity to mice, although the infection of macrophages *in vitro* was unchanged – suggesting that *Leishmania* ICP might target the CPs of the host (Besteiro *et al*., 2004). Recently, it was shown that *Entamoeba histolytica* expresses two ICP isotypes, which display different inhibitory properties against endogenous CPs and are localized in distinct compartments (Saric *et al*., 2006; Sato *et al*., 2006). Similar to the phenotype observed with *T. cruzi*, overexpression of *E. histolytica* ICPs in trophozoites led to a marked reduction of CP activity and of enzyme secretion, suggesting that ICPs also regulate endogenous CPs in this parasite (Sato *et al*., 2006).

In this study, we investigated the role of ICP in *T. brucei* by analysing parasites genetically manipulated to lack ICP. Our results suggest that *T. brucei* ICP acts as a regulator of endogenous CP activity, and thus plays a part in modulation of surface coat exchange during differentiation, intracellular proteolysis and parasite infectivity to mice.

## Results

### Targeted deletion of ICP in bloodstream form (BSF) *T. brucei*

**Fig. 1:**
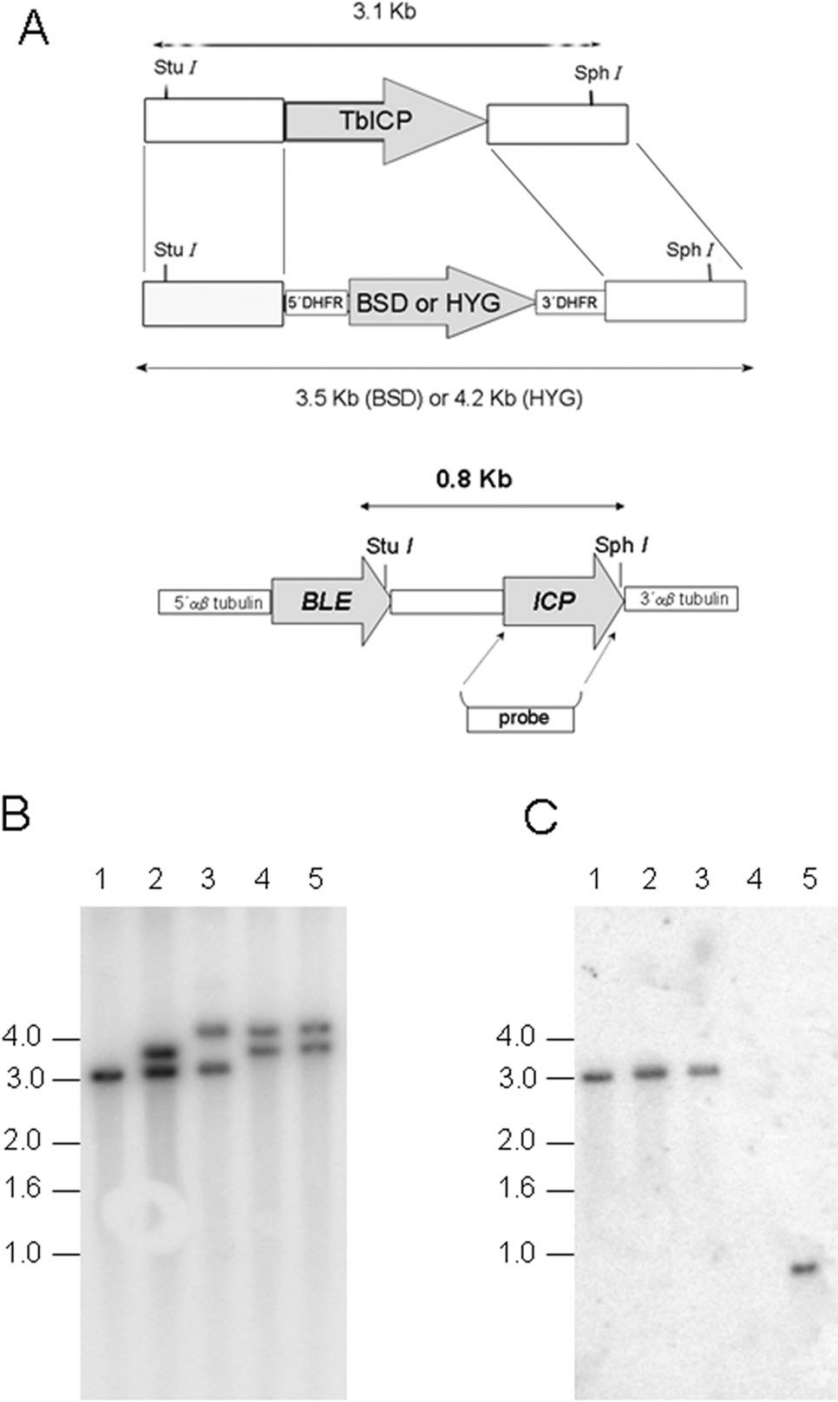
Targeted replacement of Tb*ICP* A. Schematic representation of the Tb*ICP* locus and the plasmid constructs used for gene replacement. Upper panel: ORFs are shown as arrows; intergenic and flanking DNA sequences are shown as boxes. The predicted sizes of StuI/SphI-digested DNA fragments from both native and modified Tb*ICP* locus are shown. Lower panel: Schematic representation of the re-integration of *ICP* into the tubulin locus of the Δ*icp* line. DHFR, dihydrofolate reductase gene; BSD, blasticidin-resistance gene; HYG, hygromycin-resistance gene. B and C. Southern blot analysis. Genomic DNA was digested with StuI and SphI, separated on a 0.8% agarose gel, blotted onto a nylon membrane and hybridized with ^32^P-labelled DNA probes; 5′ FR of Tb*ICP*(B) and Tb*ICP* ORF (C). Lane 1, WT *T. brucei*; lane 2, *BSD*-resistant heterozygote; lane 3, *HYG*-resistant heterozygote; lane 4, Δ*icp*; lane 5, Δ*icp:ICP*.

Targeted deletion of the diploid *T. brucei ICP* (Tb*ICP*) locus was achieved by homologous recombination. The two alleles were sequentially replaced after BSF transfection with linearized targeting constructs pGL1149 and pGL1151, containing selectable markers between *ICP* 5′ and 3′ flanking regions (FRs) (Fig. 1A). For the first allele, transfection with the pGL1151 construct yielded a population of parasites resistant to hygromycin. This population was used for the second round of transfections with the pGL1149 (blasticidin) construct, and three clones were obtained. The clones were analysed by Southern blot, one of which is presented (Fig. 1B and C). The Tb*ICP* gene was targeted into the tubulin locus of the Δ*icp* mutants to generate lines re-expressing *ICP* (designated Δ*icp*:*ICP*) (Fig. 1A, lower panel). A probe to the 5′ FR of Tb*ICP* hybridized with a 3.1 kb SphI/StuI DNA fragment containing the *ICP* gene in wild type (WT) parasites, hygromycin-resistant and blasticidin-resistant heterozygotes, but not in Δ*icp* (Fig. 1B). Probe hybridization to DNA fragments of 3.5 and 4.2 kb, corresponding to the replacement of *ICP* with the blasticidin- or the hygromycin-resistance genes, was observed in the respective heterozygotes, Δ*icp* and Δ*icp*:*ICP* (Fig. 1B). Hybridization with a probe to the coding region of Tb*ICP* revealed the presence of the gene in WT and in heterozygotes, but not in Δ*icp* (Fig. 1C). As expected, the Tb*ICP* probe hydridized to a 0.8 kb DNA fragment in the re-expressing cell line, indicating that the *ICP* gene was re-integrated into the tubulin locus (Fig. 1C).

**Fig. 2:**
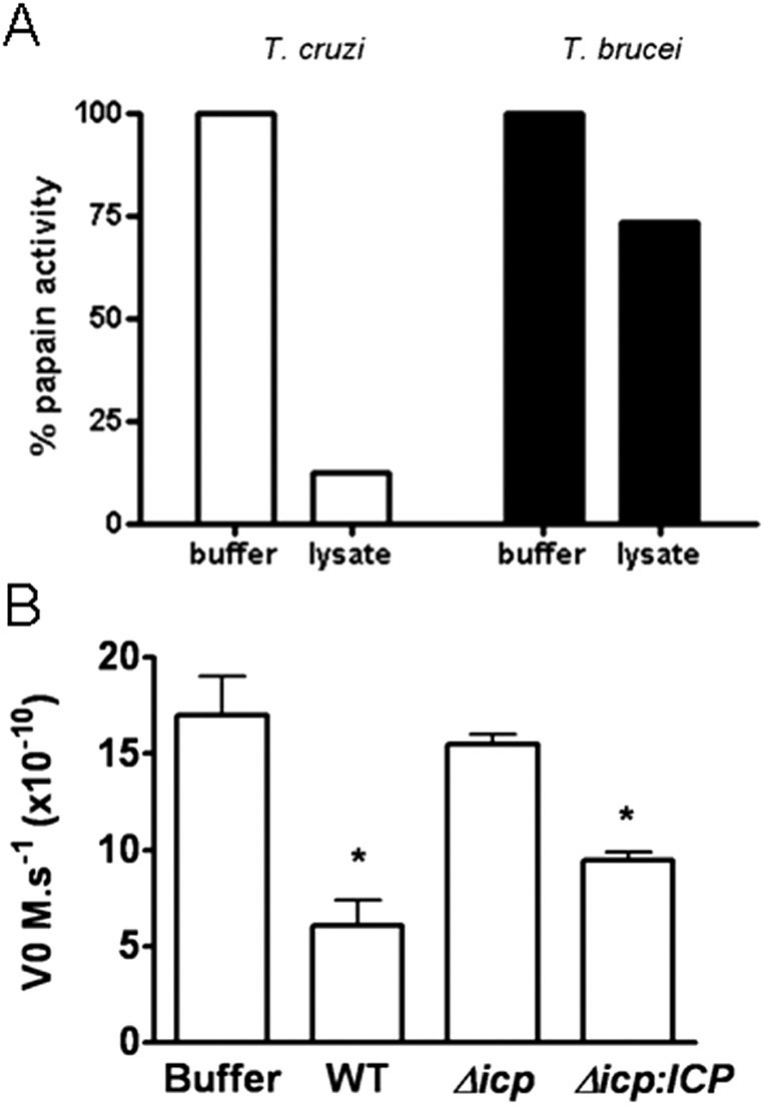
Δ*icp* lacks CP inhibitory activity A. *T. cruzi* epimastigote lysates and *T. brucei* WT BSF lysates were boiled and tested (8.5 μg protein ml^−1^) for inhibitory CP activity by pre-incubation with 3 nM papain for 20 min. The residual activity of the enzyme was measured using 15 μM Z-Phe-Arg-MCA. B. BSF lysates (50 μg protein) were boiled and tested for the inhibition of papain by pre-incubating with 2 nM papain for 20 min, followed by determination of residual peptidase activity using 5 μM of Z-Phe-Arg-MCA. Asterisk shows scores statistically significant from buffer at *P* < 0.05.

**Fig. 3:**
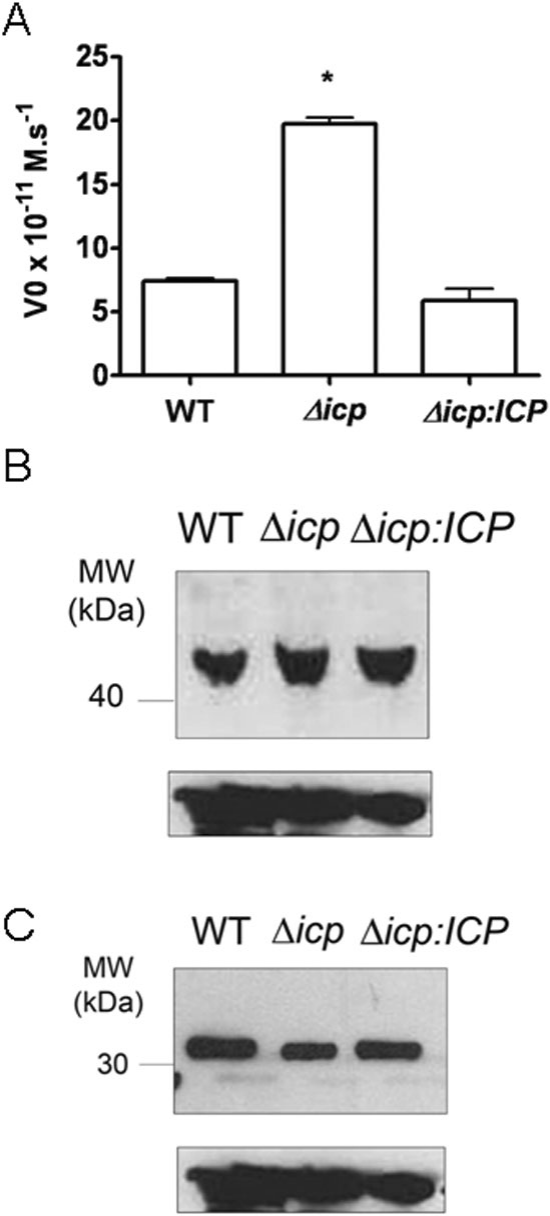
CP expression and activity. A. Five micrograms of BSF lysates was tested for peptidase activity using 5 μM of Z-Phe-Arg-MCA as a substrate. The activity sensitive to inhibition by 10 μM of E-64, which corresponds to CP activity, is shown. The experiments were performed in quadruplicate and are represented as mean values with standard deviations (SD). The analysis of significance was performed using anova, and the asterisk indicates the scores that are statistically significantly at *P* < 0.05. B and C. Western blot analysis of BSF lysates (equivalent to 5 × 10^5^ parasites per lane) using antiserum to (B) brucipain or to (C) *T. brucei* cathepsin B. Antibodies to anti-EF1α were used to visualize loading controls (bottom panels).

We were unable to detect ICP expression by Western blot analysis in WT parasites using a variety of different antisera raised against recombinant ICP. Thus, the presence of functional ICP in parasite lysates was assessed by measuring inhibition of CP activity. Taking advantage of the fact that ICP is thermostable (Monteiro *et al*., 2001), lysates were boiled in order to inactivate endogenous peptidases prior to incubation with papain. We observed that boiled lysates of *T. cruzi* inhibited papain activity more efficiently than those of WT BSF *T. brucei* (Fig. 2A). Considering that recombinant chagasin and ICP inhibit papain with similar potency (Monteiro *et al*., 2001; Sanderson *et al*., 2003), these results suggest that the levels of ICP in *T. brucei* BSF are lower than those of chagasin in *T. cruzi*. Low expression levels of ICP in *T. brucei* BSF could account for the lack of detection by Western blotting. As expected, lysates of WT and Δ*icp*:*ICP* inhibited about 60% of papain activity, while no inhibitory activity was detected in Δ*icp* lysates (Fig. 2B), even when tested at 10-fold higher concentrations (not shown), indicating that functional ICP is absent from Δ*icp*. Lysates from Δ*icp*:*ICP* inhibited papain slightly less efficiently than lysates of WT parasites (Fig. 2B), suggesting that the levels of ICP expression in the complemented line are not identical to those in WT parasites. Titration of boiled parasite lysates against papain revealed that ICP levels in Δ*icp*:*ICP* are approximately half of those in WT. We next assessed the amounts of functional CPs in lysates of BSF by enzymatic assays using fluorogenic substrates. The CP activity present in Δ*icp* lysates was threefold higher than in WT or Δ*icp*:*ICP* (Fig. 3A). The titration of CPs in the lysates of WT revealed that the CP : ICP ratio in *T. brucei* is approximately 7:1. No alteration in brucipain or cathepsin B-like CP protein expression by Western blot could be detected (Fig. 3B and C), indicating that the lack of the *ICP* did not induce changes in the expression and/or turnover of these enzymes and that the increase in CP activity was due to the absence of ICP.

### Deletion of TbICP induces alterations in parasite growth

**Fig. 4:**
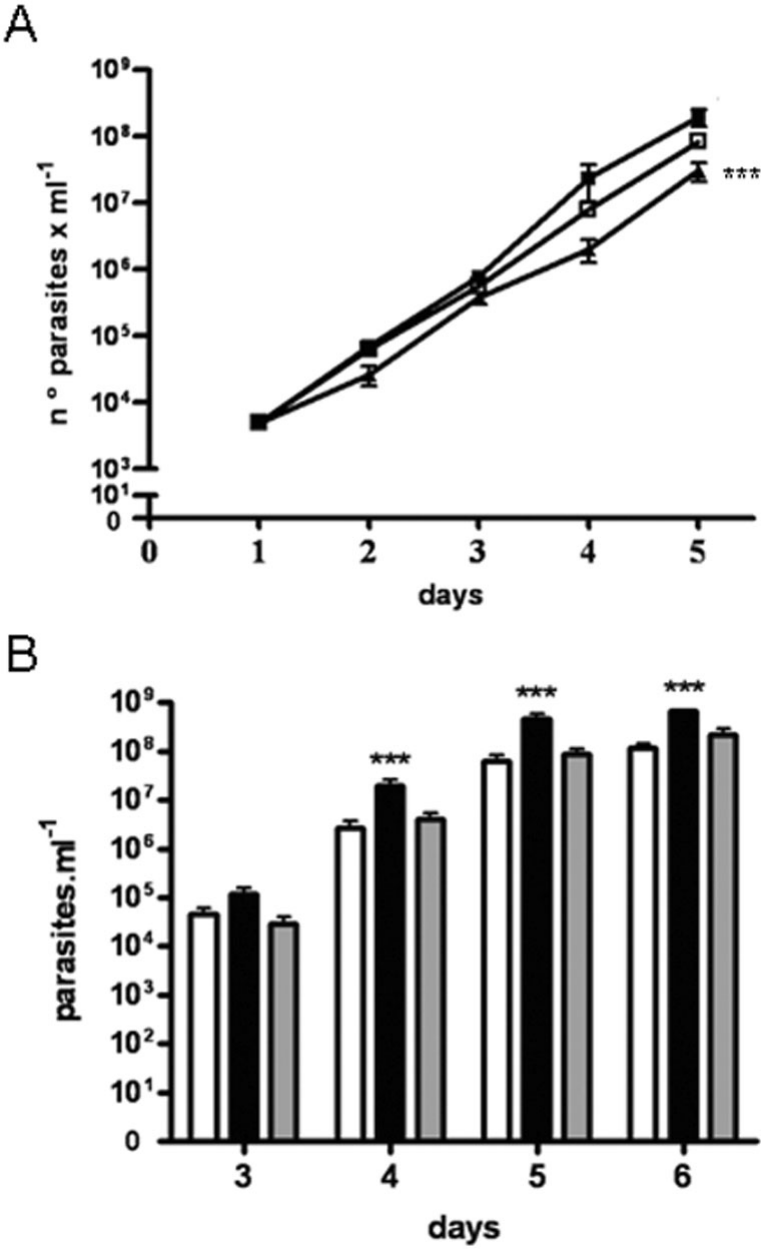
Growth of *T. brucei* A. BSF parasites were seeded at 5 × 10^3^ ml^−1^ and grown for 5 days *in vitro*, with culture density being analysed daily. At the third day, the cultures were diluted to 5 × 10^3^ ml^−1^. The mean densities at days 4 (WT: 7 × 10^5^ ml^−1^; Δ*icp*: 2.3 × 10^5^ ml^−1^; Δ*icp:ICP*: 7 × 10^5^ ml^−1^) and 5 (WT: 9.6 × 10^5^ ml^−1^; Δ*icp*: 3.5 × 10^5^ ml^−1^; Δ*icp:ICP*: 8.6 × 10^5^ ml^−1^) were multiplied by the respective dilution factors for presentation. The assay was performed in triplicate and four independent times (***P< 0.001, in comparison with WT and Δ*icp:ICP*). Keys: closed square, wild type; open square, Δ*icp:ICP*; triangle, Δ*icp*. B. BSF parasites were purified from the blood of infected mice and injected in Balb/c mice (10^3^ parasites per animal). The parasitemia was determined by counting the number of parasites in 5 μl of blood from days 3 to 6 of infection. Five mice were used per group, and the graph is representative of two independent experiments (***P < 0.001, in comparison with WT and Δ*icp:ICP*). White bars, WT parasites; black bars, Δ*icp*; grey bars, Δ*icp:ICP*.

Growth rate analysis of Δ*icp* in culture indicated that it grew more slowly than WT or Δ*icp*:*ICP* over a 5 day period (Fig. 4A), and this phenotype was reproducible in three independent Δ*icp* clones. Δ*icp* did not have apparent alterations in morphology or cell cycle progression as compared with WT, suggesting that the reduced growth rate might be due to changes in the parasite's metabolism.

Wild type and *ICP* mutant cell lines were inoculated into Balb/c mice, and parasite density in the blood was examined from day 3 to day 6 (Fig. 4B). Surprisingly, Δ*icp* parasites grew better than WT parasites *in vivo*, reaching a significantly higher parasitemia than the WT or Δ*icp*:*ICP* lines. In addition, approximately 50% of the mice infected with Δ*icp* died at day 7, while the mice infected with WT survived until day 10 post infection (not shown), indicating that deficiency in ICP increased the parasite's virulence in the mammalian host.

### Deletion of TbICP increases the resistance to a synthetic CP inhibitor

**Fig. 5:**
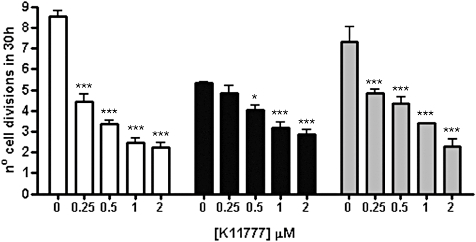
Δ*icp* has increased resistance to a CP inhibitor BSF parasites were inoculated at 5 × 10^4^ ml^−1^ in culture medium in the presence of varying concentrations of K11777, and cultivated for 2 days at 37°C. The controls were cultivated in the presence of 0.5% DMSO. The culture densities were monitored at 12, 24 and 30 h, and the numbers of cell divisions by 30 h are given. The experiments were performed in triplicate, two independent times, and are reported as mean and standard deviations of the six replicates. The analysis of significance was performed using two-way anova and the Bonferroni post-test at a significance of 5%. Single asterisks represent scores that are statistically significant at *P* < 0.05, and triple asterisks show scores statistically significant at *P* < 0.01. White bars, WT parasites; black bars, Δ*icp*; grey bars, Δ*icp:ICP*.

Synthetic CP inhibitors have been shown to kill *T. brucei* BSF in culture (Troeberg *et al*., 1999), and their trypanocidal effect was associated with the inactivation of the cathepsin L-like CP of the parasite. Considering that Δ*icp* parasites have higher CP activity, we decided to test whether that could have an impact on their sensitivity to synthetic CP inhibitors. We monitored the densities of parasite cultures in the presence or absence of the inhibitor at 12, 24 and 30 h. Because Δ*icp* grows slower *in vitro*, at the end of this period the culture density of the mutant line was about half that of WT or Δ*icp*:*ICP* in the absence of the drug. However, in the presence of 0.25 μM of N-Pip-F-hF-VSPh (K11777), the culture densities of the three lines were nearly identical (∼2 × 10^5^ ml^−1^), suggesting that the growth of WT and of Δ*icp*:*ICP*, but not that of Δ*icp*, were significantly affected by K11777. In order to verify whether Δ*icp* is refractory to the toxic effects of the drug, we calculated the number of divisions that each line had undergone in 30 h (Fig. 5). We observed that the growth of WT parasites was inhibited by 50% in the presence of 0.25 μM of K11777, while it was necessary to increase drug concentrations 4-fold (to 1 μM) to observe a similar effect in Δ*icp* parasites. Δ*icp*:*ICP* had similar drug sensitivity to that of WT parasites, confirming that the increased resistance displayed by Δ*icp* was due to lack of *ICP*. These results show that ICP levels affect BSF sensitivity to the trypanocidal effect of synthetic CP inhibitors, and suggest that ICP modulates the availability of active CPs in the parasite.

### Deletion of TbICP leads to increased degradation of anti-VSG IgG

**Fig. 6:**
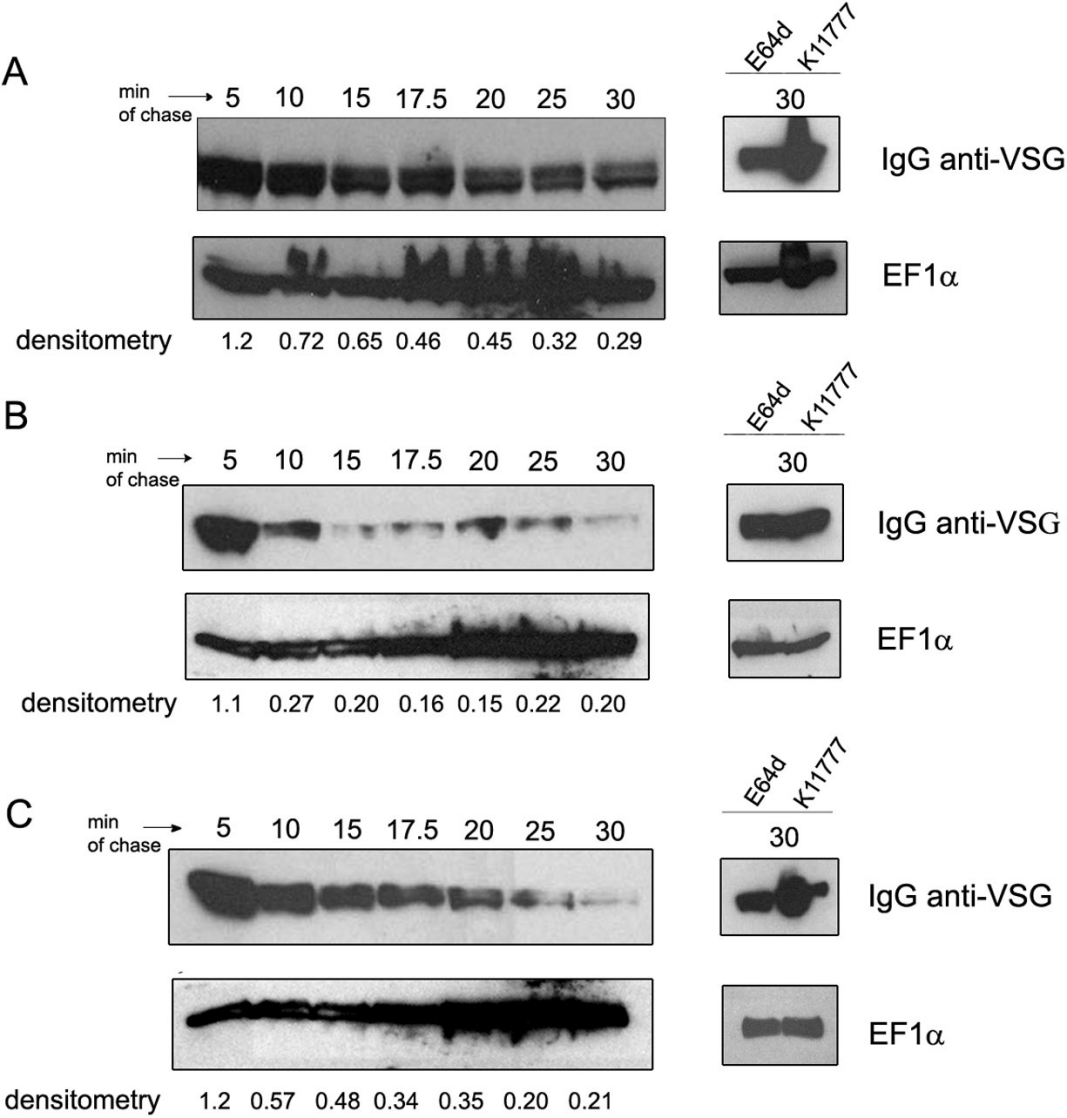
Increased degradation of anti-VSG IgG by Δ*icp*. BSF parasites were incubated for 30 min on ice with rabbit anti-VSG 221 IgG, prior to incubation at 37° for 5–30 min to detect IgG degradation (left panels). Cells were treated with 20 μM of K11777 (VSPh) or E64d prior to incubation with IgG and were chased for 30 min at 37°C (right panels). Whole-cell lysates were prepared, and equivalent of 5 × 10^6^ cells were loaded in a SDS-PAGE gel, transferred to nitrocellulose membranes, followed by incubation with anti-rabbit IgG HRP conjugate. The densitometry indicates the ratio of the intensity of IgG to EF1-α. A. WT parasites. B. Δ*icp*. C. Δ*icp:ICP*.

The surface of BSF *T. brucei* is covered by a dense coat of its main surface antigen, the variant surface glycoprotein (VSG), which is attached to the membrane via a glycosylphosphatidylinositol anchor (Ferguson, 1999). VSGs are encoded by a large family of genes/pseudogenes sequentially and uniquely expressed at a given time point, which enables trypanosomes to evade the host's immune response (Berriman *et al*., 2005). VSG is constitutively removed from the parasite's surface by rapid internalization and recycling, a process that mediates the clearance of anti-VSG antibodies from the surface and might contribute to the parasite's persistence in the immune-competent host (Seyfang *et al.*, 1990; O'Beirne *et al.*, 1998; Gruszynski *et al*., 2003; 2006; Engstler *et al*., 2004). After internalization, VSG is recycled back to the surface, while the antibodies are degraded by intracellular peptidases (O'Beirne *et al*., 1998; Pal *et al*., 2003). To address whether ICP could regulate the peptidases responsible for IgG processing in BSF, we assessed the degradation of anti-VSG antibodies following internalization (Fig. 6). In WT parasites, an approximate 50% reduction in the amount of intact IgG was detected after 20 min of chase, and the protein was significantly degraded within 30 min (Fig. 6A, upper panel, left). In contrast, the amount of elongation factor 1 (EF1), used as an endogenous control, was unchanged during the chase, showing that the reduction in IgG was not due to non-specific protein degradation during the preparation of the lysates (Fig. 6, bottom panels). The antibodies were much more rapidly degraded by Δ*icp* parasites, being reduced by 80% within the first 15 min of chase (Fig. 6B, upper panel, left), a phenotype that was partially rescued in Δ*icp*:*ICP* parasites (Fig. 6C). No difference in the internalization of transferrin-FITC was observed between the three lines (data not shown), ruling out that the differences observed in the amount of IgG detected are due to alterations in the endocytic activity of the transgenic parasites. Furthermore, pretreatment of the cells with two membrane-permeable CP inhibitors, K11777 and E64d, prevented IgG degradation (Fig. 6A–C, right panels), demonstrating that CPs are the main peptidases contributing to IgG degradation in BSF. The results indicate that the increased IgG degradation by the Δ*icp* parasites was due to higher CP activity in these parasites, suggesting that ICP modulates endogenous CP function in BSF parasites.

### Cell surface coat exchange during differentiation from BSF to procyclic forms (PCF)

**Fig. 7:**
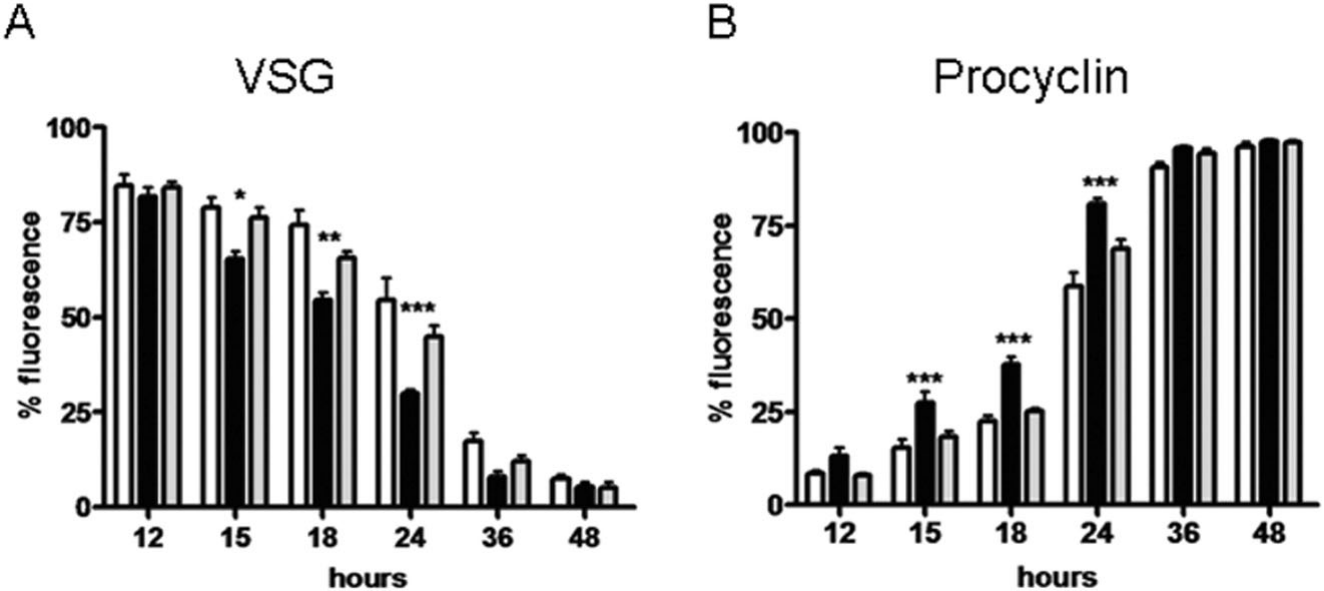
Differentiation from bloodstream to PCF. BSF were incubated in SDM-79 medium at 27°C, in the presence of 6 mM *cis*-aconitate and samples analysed at the indicated times. The exchange of the cell surface coat from VSG (A) to procyclin (B) was quantified by flow cytometry and the percentage of fluorescence cells plotted. The experiments were performed in triplicate on three separate occasions. The graph shows the means plus standard deviations of the nine replicates. The analysis of significance was performed using two-way anova and the Bonferroni post-test at a significance of 5%. Single asterisks represent scores that are statistically significant at *P* < 0.05, and double asterisks represent scores statistically significant at *P* < 0.01. White bars, WT parasites; black bars, Δ*icp*; grey bars, Δ*icp:ICP*.

One important step in the progression of infection by *T. brucei* is the transformation of short stumpy BSF to PCF after uptake by tsetse flies. The differentiation is characterized by several metabolic and morphologic changes, including the expression of cell surface stage-regulated proteins such as procyclin and the removal of the old VSG coat. BSF to PCF differentiation can be induced *in vitro* by *cis*-aconitate and low temperature (Ziegelbauer *et al*., 1990). Because previous studies have correlated CP activity with the differentiation of *T. cruzi* (Tomás and Kelly, 1996; Santos *et al*., 2005), we tested whether deletion of *ICP* would have an impact on *T. brucei* differentiation *in vitro*. During the differentiation of synchronous populations of *T. brucei* enriched in short stumpy BSF, the exchange of the VSG coat to procyclin occurs rapidly and synchronously within 4–24 h (Ziegelbauer*et al*., 1993; Van Deursen *et al*., 2001; Gruszynski *et al*., 2003; 2006). However, coat exchange during *in vitro* differentiation of exponentially growing cultures of the 427 strain was shown to occur much slower (24–48 h) and asynchronously (Roditi *et al*., 1989; Mutomba and Wang, 1998). We monitored the appearance of cell surface procyclin and VSG release by flow cytometry in the three lines during 12–48 h after the induction of differentiation by *cis*-aconitate and temperature drop (Fig. 7). We observed that a higher proportion of the Δ*icp* cell line had cell surface procyclin by 15 h of differentiation (Fig. 7A and B) than with WT parasites. The appearance of procyclin-positive cells correlated with a decrease in the number of VSG-positive cells (Fig. 7A), and loss of surface VSG was more rapid in Δ*icp*. After 18 h, about half of the Δ*icp* population had replaced VSG with procyclin, while only 20% of WT parasites had exchanged their surface coat. This phenotype was more evident at 24 h, when Δ*icp* had nearly completed surface coat exchange (Fig. 7B), while only half of WT cells were positive for procyclin in the same time period. The coat exchange of parasites re-expressing *ICP* was similar, but not identical, to that of WT cells, indicating that re-introduction of *ICP* partially complemented faster coat exchange of Δ*icp*. Importantly, the PCF Δ*icp* mutants also grew slower than WT parasites *in vitro*, ruling out the possibility that the higher number of procyclin-positive cells might have resulted from accelerated growth of the differentiated Δ*icp*.

**Fig. 8:**
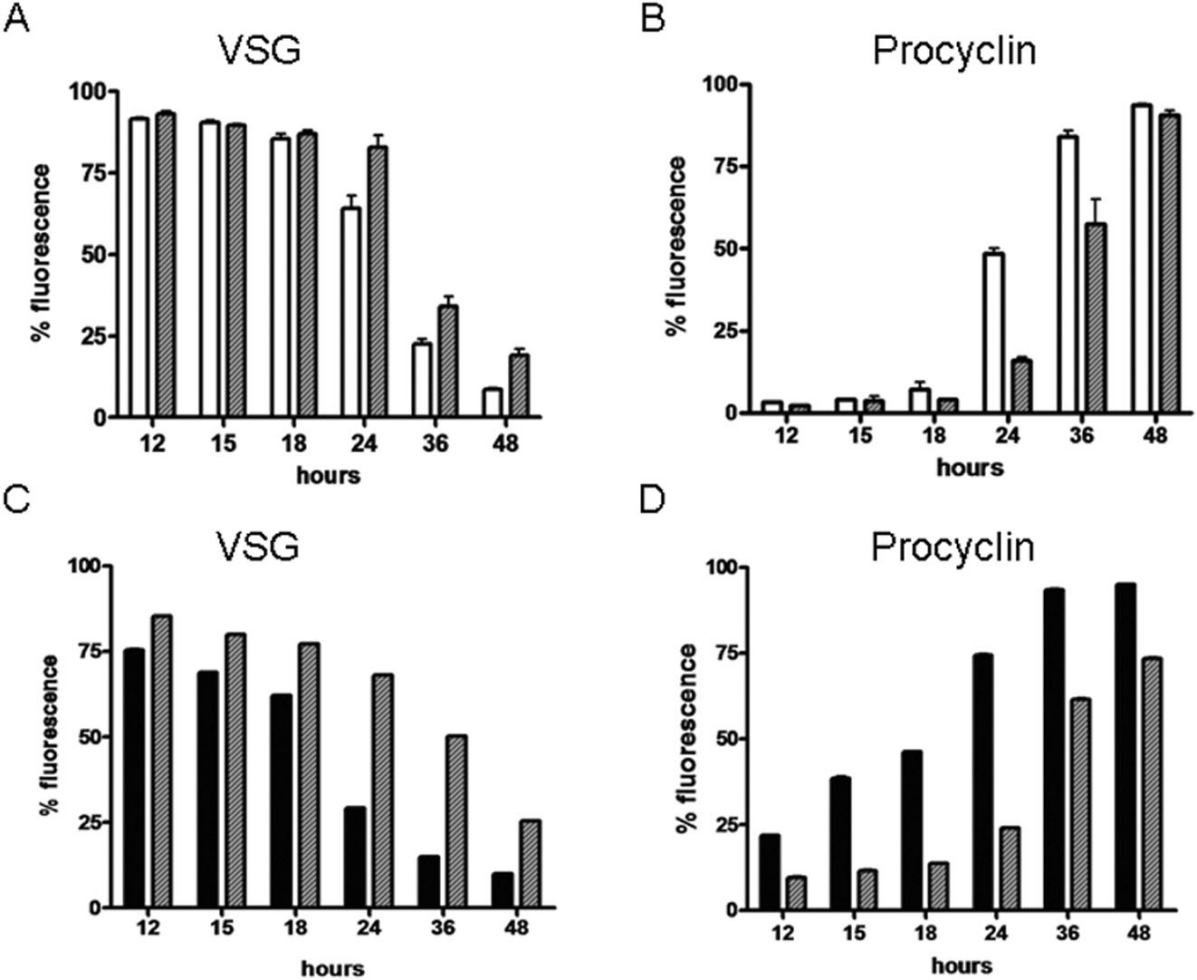
Involvement of CPs in the differentiation from BSF to PCF. Differentiation of Δ*icp* BSF to PCF in the presence of the irreversible CP inhibitor K11777 was analysed as described in Fig. 7. A and B. WT parasites. White bars, WT parasites; hatched grey bars, WT parasites + 0.25 μM K11777. C and D. Δ*icp*. Black bars, Δ*icp*; hatched grey bars, Δ*icp*+ 2 μM K11777.

It was recently reported that the tyrosine phosphatase TbPTP1 plays a pivotal role in controlling BSF to PCF differentiation (Szoor *et al*., 2006). A cell-permeable inhibitor of this enzyme, BZ3, induced differentiation of a small subset of BSF parasites in populations grown in asynchronous cultures. It was suggested that the cells sensitive to BZ3-induced differentiation were committed to early events in stumpy formation before morphological differentiation occurred, being defined as stumpy* (Tasker *et al*., 2000). We treated BSF with BZ3 and accessed procyclin appearance in early time points (3–12 h) as a way to assess whether Δ*icp* populations were enriched in stumpy* forms, thus accounting for the more efficient coat exchange observed in *cis*-aconitate-induced differentiation. The percentage of procyclin-positive cells was low (< 15%) in the three parasite lines at 12 h (data not shown). Furthermore, although there was a small increase in the proportion of procyclin-positive cells within the Δ*icp* population at 3 h (4% in Δ*icp* versus 1.5% in WT parasites), all three parasite lines had equivalent proportions of procyclin-positive cells by 6 h, indicating that faster coat exchange displayed by Δ*icp* parasites could not be attributed to a higher proportion of stumpy* forms prior to triggering of differentiation with *cis*-aconitate. Importantly, treatment of parasites with K11777 during differentiation significantly delayed coat exchange of WT parasites (Fig. 8A and B) and of Δ*icp* (Fig. 8C and D), confirming that CP activity contributes to the efficiency of coat exchange during *T. brucei* differentiation. Notably, the effect of the synthetic CP inhibitor in delaying the coat exchange was detected with WT parasites only by 24 h (Fig. 8A and B), while this effect was observed with Δ*icp* parasites in the first 12 h (Fig. 8C and D). This further suggests that the accelerated coat exchange in the mutants was associated with increased CP activity. The parasites remained intact and mobile in the presence of the inhibitor during the assay, arguing against non-specific effects due to toxicity of the drug. Taken together, these results suggest that ICP plays a role in controlling the differentiation process through the modulation of endogenous CP activity.

## Discussion

We have used target gene deletion as a strategy to investigate the function of the chagasin-like CP inhibitor, ICP, in *T. brucei*. Deletion of *ICP* led to increased CP activity in lysates of BSF parasites, while the expression levels of brucipain and of the cathepsin B-like peptidase were unchanged. In WT *T. brucei*, the CP : ICP ratio calculated by titration experiments revealed that CPs are in sevenfold excess, confirming that ICP is expressed at much lower levels than the CPs. Comparative analyses of papain inhibition by boiled lysates from different parasite species indicated that lysates of *T. brucei* have lower papain-inhibitory activity than those of *T. cruzi*. As recombinant TbICP is thermo-resistant and displays high affinity for papain (Sanderson *et al*., 2003), these observations suggest that ICP is expressed at low levels in *T. brucei* compared with chagasin expression in *T. cruzi* (Monteiro *et al*., 2001; Besteiro *et al*., 2004; Santos *et al*., 2005). Low expression levels might explain why we were unable to detect ICP in parasite lysates by Western blot. Nonetheless, lack of ICP led to a 3- to 4-fold increase in total CP activity, suggesting that, as observed in *T. cruzi* and in *Entamoeba*, changes in inhibitor expression have a significant impact on the overall CP content in parasites despite the unfavourable inhibitor–enzyme ratio (Santos *et al*., 2005; Sato *et al*., 2006). Because conversion of zymogens of the CPs to active forms is thought to occur by auto-catalysis, the rate of CP zymogen processing and/or sorting could be altered upon ICP deletion, which could be the explanation for these observations.

Studies with synthetic CP inhibitors have previously suggested that cathepsin L-like peptidases are the main targets of these compounds in trypanosomes and in *Leishmania.* Our analyses of the effect of K11777 on the growth of BSF showed that Δ*icp* displays fourfold greater resistance to this drug than WT parasites, a phenotype that could be explained by increased availability of free CPs. The fourfold increase in resistance observed correlates well with the increase in peptidase activity encountered in the Δ*icp* (3 fold), arguing in favour of the hypothesis that a higher amount of the synthetic drug is required to inactivate free CPs in Δ*icp* parasites. Furthermore, it suggests that the availability of active CPs is subject to endogenous control by ICP. Although the mechanism by which synthetic CP inhibitors cause *T. brucei* death is unknown, in *T. cruzi* they promote accumulation of CP zymogens in the Golgi, causing disruption of the intracellular traffic and abnormalities in the secretory pathway (Engel *et al*., 1998). The isolation of resistant *T. cruzi* epimastigote populations under selective pressure revealed that upregulation of exocytosis promoted the secretion of unprocessed CP precursors, sparing the cells from the deleterious effect of the inhibitor (Engel *et al*., 2000). Intriguingly, chagasin overexpression in *T. cruzi* also increased parasite resistance to the same drug (Santos *et al*., 2005). Although the mechanisms underlying the increase in resistance are unclear, parasites overexpressing chagasin display an increase in the secretion of CP precursors to the flagellar pocket (C.C. Santos and A.P.C.A. Lima, unpubl. data), which might play some part in the resistance. These observations suggest that the balance of CPs and endogenous inhibitors can affect sensitivity to CP inhibitors in multiple ways.

In mammals, *T. brucei* BSF transform from slender proliferating forms into stumpy non-proliferating forms, which are competent to differentiate to PCF once ingested by the tsetse fly (Turner *et al.*, 1995). Our results showed accelerated coat exchange in conditions of *cis*-aconitate-induced differentiation in Δ*icp*, and this phenotype was reverted in the presence of synthetic CP inhibitors, suggesting that more efficient coat exchange is mediated by CP activity. Importantly, the use of the tyrosine phosphatase inhibitor BZ3 enabled us to verify that the proportion of cells committed to differentiate prior to induction was similar in the three lines, suggesting that CPs are fulfilling their function at steps subsequent to those that trigger differentiation. After triggering, BSF-specific proteins must be degraded (or released) and significant changes in cell shape occur. It is plausible that lysosomal CPs are required for the massive protein degradation and remodelling that occurs during differentiation, their activity being subjected to regulation by ICP.

The inhibition of VSG release during differentiation by treatment with synthetic CP inhibitors suggests that CPs play a role in surface coat remodelling either directly or indirectly, and provides evidence that ICP negatively modulates endogenous CP activity in BSF. Two main mechanisms are thought to govern VSG release: (i) GPI hydrolysis mediated by an endogenous GPI-phospholipase C (GPI-PLC), responsible for constitutive shedding in exponentially growing BSF; and (ii) endoproteolysis mediated by a zinc metallopeptidase (MSP-B) that is upregulated during differentiation (Bangs *et al*., 1997; Gruszynski *et al*., 2003; 2006). Proteolytic release of VSG occurs via truncations upstream of the C-terminal anchor, but the cleavage sites differ between VSG variants (Bangs *et al*., 1997). Endoproteolysis is the main pathway responsible for VSG release during differentiation, and the concerted action of both GPI-PLC and MSP-B are thought to mediate complete shedding of the old VSG coat (Gruszynski *et al*., 2006). Despite the fact that inhibitors of metallopeptidases significantly block the proteolytic release of VSG during *in vitro* differentiation of stumpy BSF, it was observed that incubation of parasites with the membrane-permeable synthetic CP inhibitor Mu-F-hF-BzPr resulted in a small but noticeable reduction of VSG release (Gruszynski *et al*., 2003). In addition, a previous independent study using the same CP inhibitor during differentiation of the 427 strain showed that VSG was retained at the surface of PCF parasites, suggesting that CPs might play a role in the release of the VSG coat (Mutomba and Wang, 1998). It is possible that the relative contribution of CPs to VSG release varies among different parasite strains.

During *T. brucei* infections, a robust immune response is raised against VSG, which is mainly evaded by the parasite due to VSG antigenic variation. In addition, *in vitro* studies have shown that anti-VSG antibodies bound to the parasite surface are rapidly internalized and degraded, while VSG remains intact (O'Beirne *et al*., 1998). Although it has not yet been demonstrated that IgG degradation plays a role in immune evasion *in vivo*, it was proposed that it could make a contribution to the prevention of antibody-dependent destruction of the parasite. We observed that treatment of BSF with membrane-permeable CP inhibitors significantly blocked anti-VSG IgG degradation, indicating that these peptidases play a central role in IgG degradation. In agreement with this, Δ*icp* parasites were capable of degrading anti-VSG IgG with much higher efficiency than WT parasites, providing additional evidence that ICP negatively controls CP-mediated intracellular proteolysis in *T. brucei*. Brucipain is upregulated in stumpy forms (Pamer *et al.*, 1989; Caffrey *et al*., 2001), and these forms are more resistant to antibody-mediated lysis and differentiate more efficiently than monomorphic forms. This is consistent with our findings that increased CP activity in BSF promotes enhanced IgG degradation and cell differentiation.

In BSF, it was shown that degradation of fluorescein-coupled IgG, measured by changes in fluorescence after internalization, is abolished when the internal pH of endosomal compartments are raised (Pal *et al*., 2003), supporting the notion that IgG degradation requires the action of peptidases found in an acidic compartment, such as the lysosome. The acidic cathepsin L-like CP of *T. brucei* (brucipain) is located in the parasite's lysosome (Caffrey *et al*., 2001), and we have previously shown that this peptidase is the main target of the synthetic inhibitor K11777 in *T. gambiense* (Nikolskaia *et al*., 2006). Considering that K11777 blocks IgG degradation in *T. brucei*, it is possible that brucipain, and not the cathepsin B-like peptidase, is the main CP mediating IgG degradation in BSF. Of note, it was reported that potent inhibitors of brucipain Z-Phe-Tyr(OtBu)-CHN_2_ and Z-Phe-Tyr-CHO inhibit lysosomal proteolysis of transferrin, further suggesting that brucipain is largely responsible for lysosomal proteolysis in BSF (Nkemgu *et al*., 2003). On the other hand, recent studies employing RNAi to address the roles of brucipain and of the cathepsin B-like enzyme in *T. brucei* suggested that the latter is responsible for transferrin degradation (Mackey *et al*., 2004). In view of these findings, we postulate that ICP modulates intracellular proteolysis by means of inactivating brucipain and the cathepsin B-like CP in lysosomes. The relative contribution of ICP in the regulation of each individual CP remains to be investigated. Although the inhibition of *T. brucei* CPs by ICP has not been studied at the biochemical level, it is known that the affinity of recombinant ICP for human cathepsin B is approximately 5-fold higher than that for human cathepsin L (Sanderson *et al*., 2003). If a similar inhibition pattern occurs with regards to the parasite CPs, it is possible that ICP could interact with both CPs of *T. brucei in vivo*.

Finally, we observed that lack of ICP enhanced parasite virulence *in vivo*, as observed by higher parasitemia in the blood of infected mice and a more rapid onset of death in the animals. Although not directly demonstrated, it is very likely that increased levels of endogenous CPs in the null mutants are responsible for increased virulence. This hypothesis is in agreement with several studies showing that *T. brucei* CP activity is required for optimal parasite survival *in vitro* and *in vivo* (Troeberg *et al*., 1999; 2000; Greenbaum *et al*., 2004; Fujii *et al*., 2005; Vicik *et al*., 2006). The precise biological processes requiring the action of CPs for parasite survival are not fully understood. Even though Δ*icp* is potentially capable of clearing anti-parasite IgG more efficiently than WT parasites, it is unlikely that antibodies play a major role in eliminating parasites during the early stages (3–6 days) of infection. Rather, macrophages are thought to play a protective role during this phase of infection – clearing trypanosomes by phagocytosis and/or by secreting TNF-α and nitric oxide (NO), which are trypanolytic and trypanostatic (Magez *et al*., 1997; Gobert *et al*., 1998; Tabel *et al.*, 1999). It has been reported that cruzipain modulates the activation of murine macrophages, downregulating the induction of NO synthase and promoting increased survival of *T. cruzi* (Stempin *et al*. 2002). Thus, it is tempting to speculate that *T. brucei* CPs also play a role in the interaction of BSF and macrophages (and/or other cells of innate immunity), ultimately contributing to increased parasite numbers during early infection. In addition, we have recently demonstrated that CPs are directly involved in the traversal of the blood–brain barrier by *T. b. gambiense*, revealing an unexpected role of these enzymes in brain pathology (Nikolskaia *et al*., 2006). By attenuating parasite virulence, ICP expression might be beneficial for the long-term survival of the parasites in their natural hosts.

## Experimental procedures

### Constructs for the deletion of ICP

The 5′ and 3′ FRs of the *T. brucei ICP* gene (Tb927.8.6450) were obtained by polymerase chain reaction (PCR) using the primers OL1609 (CGGCGGCCGCGGTGGAGATTAAAAAAAGAAAAAAGTG)/OL1610 (CGTCTAGAGCAACAAAAATCAATGACATG) and OL1611 (CGGGGCCCGGTATGTGGAAGTGGAGAAG)/OL1612 (CGGGGCCC GATATCGGCGGGATGGAGTAAACATA) respectively, with genomic DNA of *T. brucei* EATRO795 as the template. The PCR products were cloned in the TOPO vector for sequencing. The 5′ and 3′ FRs were cloned respectively into the NotI/XbaI and ApaI sites flanking the blasticidin-resistance gene, generating vector pGL1149, or into the same sites flanking the hygromycin-resistance gene, generating vector pGL1151. For re-expression of the *T. brucei ICP* gene, the open reading frame (ORF) was obtained by PCR using the primers NT90/NT91 (Sanderson *et al*., 2003) and cloned into the vector containing a αβ-tubulin intergenic region and a phleomycin-resistance gene (Helms *et al*., 2006).

### Culturing and transfection of parasites

Bloodstream form *T. brucei* 427 was cultured at 37°C, in a 5% CO_2_ humidified atmosphere, in HMI-9 medium (Hirumi and Hirumi, 1989) supplemented with 10% (v/v) FCS and 10% (v/v) serum plus. Twenty micrograms of linearized DNA was used to transfect 5 × 10^7^ mid-log phase parasites in 0.5 ml ZPFMG (132 mM NaCl, 8 mM Na_2_HPO_4_, 1.5 mM KH_2_PO_4_, 0.5 mM magnesium acetate, 0.09 mM calcium acetate, pH 7.0, supplemented with 55 mM glucose, pH 7.0) in a 0.4 cm pulse cuvette set at 1.2 kV, 25 μF, 1 pulse. After overnight recovery, selection of clones was performed by limiting dilution with appropriate antibiotics (5 μg ml^−1^ blasticidin, 5 μg ml^−1^ hygromycin or 2.5 μg ml^−1^ phleomycin) in 24-well plates.

### Analysis of the transfectants

The genomic DNA from the tranfectants was isolated using the DNeasy kit (Qiagen) to check for the correct integration of the constructions. A Southern blot was performed using 3 μg of gDNA digested with SphI/StuI overnight at 37°C, electrophoresed in a 0.8% agarose gel and blotted onto Hybond N^+^ membrane (Amersham Pharmacia). The membrane was blocked with 1 M NaCl/1% SDS/100 μg ml^−1^ Salmon DNA sperm at 65°C for 1 h, and subsequently hybridized with a 779 bp 5′ FR Tb*ICP* or 348 bp Tb*ICP* as probes, labelled with the random primer kit (Amersham Pharmacia) overnight at the same temperature. The membrane was washed three times with 0.2× SSC/0.1% SDS for 15 min and exposed overnight.

### Enzymatic assays

Parasites were washed and resuspended in 50 mM sodium acetate, 200 mM NaCl, 5 mM EDTA (pH 5.5), 1% NP-40, incubated on ice for 10 min, followed by centrifugation at 10 000 *g* for 5 min. The protein concentration of the soluble fraction was determined using the Dc-Protein kit (Bio-Rad). Samples of 5 μg protein ml^−1^ lysates were tested for peptidase activity in 50 mM sodium acetate (pH 5.5), 200 mM NaCl, 5 mM EDTA, and 5 mM DTT using 5 μM of Z-Phe-Arg-MCA as substrate. The initial rates were calculated by linear regression of the substrate hydrolysis curves. The activities sensitive to inhibition by 10 μM of E64 are shown in Fig. 6. Because ICP is highly thermo-stable, the detection of inhibitory activity of TbICP was performed after boiling the lysates (2 mg ml^−1^) for 20 min in order to inactivate endogenous CPs, followed by recovery of the soluble fraction by centrifugation at 10 000 *g* for 10 min. The presence of inhibitory activity was checked by incubation with papain at the concentrations indicated in the legend to Figs 2A and 2B, in 50 mM Na_2_PO_4_, 100 mM NaCl, 5 mM EDTA (pH 6.5), and 2.5 mM DTT, for 20 min at room temperature. The remaining activity was measured by addition of Z-Phe-Arg-MCA at a final concentration of 5 or 15 μM. For the titration of ICP, parasite lysates were normalized for protein concentration and boiled for 20 min. The soluble fraction was recovered, and different amounts (5 independent points) were incubated with 1 nM of papain as described previously (Monteiro *et al*., 2001). The residual enzyme activity was measured by addition of Z-Phe-Arg-MCA, and the initial velocities were calculated from linear regression of the substrate hydrolysis plot. The equation of the linear regression of the V_0_ (Y) versus lysate concentration (X) plot was used to calculate the *X*-value to which Y = 0 (Monteiro *et al*., 2001).

### Analysis of parasite growth *in vitro*

Bloodstream form WT, Δ*icp* and Δ*icp*:*ICP* were inoculated into HMI-9 medium supplemented with 10% (v/v) FCS and 10% (v/v) serum plus at a concentration of 5 × 10^3^ cells ml^−1^. The parasites were cultivated for 5 days, with dilution at the third day to 5 × 10^3^ ml^−1^, and the growth was estimated by daily counts of the culture using a haemocytometer chamber. The culture densities at days 4 and 5 were multiplied by the day 3 dilution factor before being plotted for the growth curve. The experiments were performed in triplicate. The analysis of significance was performed by anova using GraphPad Prism 4.0, using the Bonferroni post-test comparing all pairs of columns at a significance of 5%.

### Sensitivity to N-Pip-F-hF-VSPh

Bloodstream forms were inoculated at 5 × 10^4^ ml^−1^ in HMI-9 containing 10% (v/v) FCS and 10% (v/v) serum plus supplemented with 0.5% DMSO or 0.5% DMSO and variable concentrations of the synthetic irreversible cysteine peptidase inhibitor *N*-methylpiperazine-urea-phe-homophe-vinylsulphone-benzene (K11777). The growth was determined by counting the cell density at 12, 24 and 30 h, using a Beckman coulter counter. The experiments were performed in triplicate on three separate occasions. The density of each culture at 30 h was used to calculate the number of divisions that had occurred. The IC_50_ was determined for the three parasite lines. The analysis of significance was performed by two-way anova using GraphPad Prism 4.0, using the Bonferroni post-test comparing all pairs of columns (all groups to each other) at a significance of 5%.

### Anti-VSG 221 IgG degradation

Bloodstream form parasites were harvested at mid-log phase of growth and labelled with anti-VSG 221 antibodies on ice for 30 min in HMI-9 at a concentration of 1 × 10^7^ ml^−1^. Parasites were then washed three times in ice-cold serum-free HMI-9 and incubated at 37°C for 5, 10, 15 or 30 min. Following the incubation period, samples were prepared for Western blot analysis. Rabbit anti-VSG IgG was detected directly using an anti-rabbit IgG-HRP conjugate (Promega) and visualized by addition of SuperSignal West Pico Chemiluminescence substrate (Pierce). For the densitometry, the bands were selected using the Scion Image Program. The intensity of EF1-α was considered 100% for each lane, and the ratio of the intensities of IgG and EF1-α was calculated; the densitometry values are indicated at the bottom of each lane in Fig. 6.

### Differentiation to the PCF

Late-log bloodstream forms (8–10 × 10^5^ ml^−1^) were harvested and suspended in warm SDM-79 medium containing 10% (v/v) FCS at a final density of 2 × 10^6^ ml^−1^. Six micromolars of *cis*-aconitate (Sigma) was added, and the cultures were incubated at 27°C to allow the differentiation. Aliquots were taken at different time periods (12, 15, 18, 24, 36 and 48 h), fixed in 2% paraformaldehyde, and analysed for the presence of VSG or procyclin at the surface by FACS using anti-VSG 221 and anti-procyclin Mab antibodies (Cedarlane Laboratories, Ontario, Canada), both diluted 1:1000 in PBS containing 1 mg ml^−1^ BSA, followed by incubation with IgG anti-rabbit or IgG anti-mouse Alexa 488 secondary antibodies respectively. The experiments were performed in triplicate on three separate occasions. For BZ3 inhibition assays, bloodstream form parasites were exposed to 150 μM PTP1B inhibitor BZ3 (Calbiochem) in SDM-79 medium containing 10% (v/v) FCS for 3, 6, 9 or 12 h. The expression of procyclin was assayed by flow cytometry using anti-procyclin Mab antibody (1:1000). K11777 was used at the IC_50_ concentrations for each line.

### Mice infections

Cultured bloodstream form parasites (1 × 10^5^) were inoculated intraperitoneally in Balb/c mice, harvested from blood after 5 days of infection, and used to inoculate intraperitoneally five mice per group at 1 × 10^3^ parasites per animal. The subsequent parasitemia was determined by counting the number of parasites in 5 μl of blood samples taken on days 3–6 of infection.

## References

[b1] Abrahamson M, Alvarez-Fernandez M, Nathanson CM (2003). Cystatins. Biochem Soc Symp.

[b2] Bangs JD, Ransom DM, McDowell MA, Brouch EM (1997). Expression of bloodstream variant surface glycoproteins in procyclic stage *Trypanosoma brucei*: role of GPI anchors in secretion. EMBO J.

[b3] Berriman M, Ghedin E, Hertz-Fowler C, Blandin G, Renauld H, Bartholomeu DC (2005). The genome of the African trypanosome *Trypanosoma brucei*. Science.

[b4] Besteiro S, Coombs GH, Mottram JC (2004). A potential role for ICP, a leishmanial inhibitor of cysteine peptidases, in the interaction between host and parasite. Mol Microbiol.

[b5] Caffrey CR, Hansell E, Lucas KD, Brinen LS, Hernandez AA, Cheng JN (2001). Active site mapping, biochemical properties and subcellular localization of rhodesain, the major cysteine protease of *Trypanosoma Brucei Rhodesiense*. Mol Biochem Parasitol.

[b6] Engel JC, Doyle PS, Palmer J, Hsieh I, Bainton DF, McKerrow JH (1998). Cysteine protease inhibitors alter Golgi complex ultrastructure and function in *Trypanosoma cruzi*. J Cell Sci.

[b7] Engel JC, Torres Garcia C, Hsieh I, Doyle PS, McKerrow JH (2000). Upregulation of the secretory pathway in cysteine proteinase inhibitor-resistant *Trypanosoma cruzi*. J Cell Sci.

[b8] Engstler M, Thilo L, Weise F, Grünfelder CG, Schwarz H, Boshart M, Overath P (2004). Kinetics of endocytosis and recycling of the GPI-anchored variant surface glycoprotein in *Trypanosoma brucei*. J Cell Sci.

[b9] Ferguson MAJ (1999). The structure, biosynthesis and functions of glycosylphosphatidylinositol anchors, and the contributions of trypanosome research. J Cell Sci.

[b10] Figueiredo da Silva AA, Vieira LDC, Krieger MA, Goldenberg S, Zanchin NIT, Guimaraes BG (2007). Crystal structure of chagasin, the endogenous cysteine-protease inhibitor from *Trypanosoma cruzi*. J Struct Biol.

[b11] Fujii N, Mallari JP, Hansell EJ, Mackey Z, Doyle P, Zhou YM (2005). Discovery of potent thiosemicarbazone inhibitors of rhodesain and cruzain. Bioorg Med Chem Lett.

[b12] Gobert AP, Semballa S, Daulouede S, Lesthelle S, Taxile M, Veyret B, Vincendeau P (1998). Murine macrophages use oxygen- and nitric oxide-dependent mechanisms to synthesize *S*-nitroso-albumin and to kill extracellular trypanosomes. Infect Immun.

[b13] Greenbaum DC, Mackey Z, Hansell E, Doyle P, Gut J, Caffrey CR (2004). Synthesis and structure-activity relationships of parasiticidal thiosemicarbazone cysteine protease inhibitors against *Plasmodium falciparum*, *Trypanosoma brucei*, and *Trypanosoma cruzi*. J Med Chem.

[b14] Gruszynski AE, DeMaster A, Hooper NM, Bangs JD (2003). Surface coat remodeling during differentiation of *Trypanosoma brucei*. J Biol Chem.

[b15] Gruszynski AE, van Deursen FJ, Albareda MC, Best A, Chaudhary K, Cliffe LJ (2006). Regulation of surface coat exchange by differentiating African trypanosomes. Mol Biochem Parasitol.

[b16] Helms MJ, Ambit A, Appleton P, Tetley L, Coombs GH, Mottram JC (2006). Bloodstream form *Trypanosoma brucei* depend upon multiple metacaspases associated with RAB11-positive endosomes. J Cell Sci.

[b17] Hirumi H, Hirumi K (1989). Continuous cultivation of *Trypanosoma brucei* blood stream forms in a medium containing a low concentration of serum-protein without feeder cell-layers. J Parasitol.

[b18] Ivens AC, Peacock CS, Worthey EA, Murphy L, Aggarwal G, Berriman M (2005). The genome of the kinetoplastid parasite, *Leishmania major*. Science.

[b19] Lonsdale-Eccles JD, Grab DJ (1987). Lysosomal and non-lysosomal peptidyl hydrolases of the blood stream forms of *Trypanosoma Brucei Brucei*. Eur J Biochem.

[b20] Mackey ZB, O'Brien TC, Greenbaum DC, Blank RB, McKerrow JH (2004). A cathepsin B-like protease is required for host protein degradation in *Trypanosoma brucei*. J Biol Chem.

[b21] Magez S, Geuskens M, Beschin A, del Favero H, Verschueren H, Lucas R (1997). Specific uptake of tumor necrosis factor-alpha is involved in growth control of *Trypanosoma brucei*. J Cell Biol.

[b22] Monteiro ACS, Abrahamson M, Lima APCA, Vannier-Santos MA, Scharfstein J (2001). Identification, characterization and localization of chagasin, a tight-binding cysteine protease inhibitor in *Trypanosoma cruzi*. J Cell Sci.

[b23] Mutomba MC, Wang CC (1998). The role of proteolysis during differentiation of *Trypanosoma brucei* from the bloodstream to the procyclic form. Mol Biochem Parasitol.

[b24] Nikolskaia OV, Lima APCA, Kim VY, Lonsdale-Eccles JD, Toshihide F, Scharfstein J, Grab DJ (2006). Blood–brain barrier traversal by African trypanosomes requires calcium signaling induced by parasite cysteine protease. J Clin Invest.

[b25] Nkemgu NJ, Grande R, Hansell E, McKerrow JH, Caffrey CR, Steverding D (2003). Improved trypanocidal activities of cathepsin L inhibitors. Int J Antimicrob Agents.

[b26] O'Beirne C, Lowry CM, Voorheis HP (1998). Both IgM and IgG anti-VSG antibodies initiate a cycle of aggregation-disaggregation of bloodstream forms of *Trypanosoma brucei* without damage to the parasite. Mol Biochem Parasitol.

[b27] Pal A, Hall BS, Jeffries TR, Field MC (2003). Rab5 and Rab11 mediate transferrin and anti-variant surface glycoprotein antibody recycling in *Trypanosoma brucei*. Biochem J.

[b28] Pamer EG, So M, Davis CE (1989). Identification of a developmentally regulated cysteine protease of *Trypanosoma brucei*. Mol Biochem Parasitol.

[b29] Pandey KC, Singh N, Arastu-Kapur S, Bogyo M, Rosenthal PJ (2006). Falstatin, a cysteine protease inhibitor of *Plasmodium falciparum*, facilitates erythrocyte invasion. PLoS Pathogens.

[b30] Riekenberg S, Witjes B, Saric M, Bruchhaus I, Scholze H (2005). Identification of EhICP1, a chagasin-like cysteine protease inhibitor of *Entamoeba histolytica*. FEBS Lett.

[b31] Rigden DJ, Mosolov VV, Galperin MY (2002). Sequence conservation in the chagasin family suggests a common trend in cysteine proteinase binding by unrelated protein inhibitors. Protein Sci.

[b32] Roditi I, Schwarz H, Pearson TW, Beecroft RP, Liu MK, Richardson JP (1989). Procyclin gene-expression and loss of the variant surface glycoprotein during differentiation of *Trypanosoma brucei*. J Cell Biol.

[b33] Sajid M, McKerrow JH (2002). Cysteine proteases of parasitic organisms. Mol Biochem Parasitol.

[b34] Salmon D, do Aido-Machado R, Diehl A, Leidert M, Schmetzer O, de A Lima AP (2006). Solution structure and backbone dynamics of the *Trypanosoma cruzi* cysteine protease inhibitor chagasin. J Mol Biol.

[b35] Sanderson SJ, Westrop GD, Scharfstein J, Mottram JC, Coombs GH (2003). Functional conservation of a natural cysteine peptidase inhibitor in protozoan and bacterial pathogens. FEBS Lett.

[b36] Santos CC, Sant'Anna C, Terres A, Cunha-e-Silva N, Scharfstein J, de AL (2005). Chagasin, the endogenous cysteine protease inhibitor of *Trypanosoma cruzi*, modulates parasite differentiation and invasion of mammalian cells. J Cell Sci.

[b37] Saric M, Vahrmann A, Bruchhaus I, Bakker-Grunwald T, Scholze H (2006). The second cysteine protease inhibitor, EhICP2, has a different localization in trophozoites of *Entamoeba histolytica* than EhICP1. Parasitol Res.

[b38] Sato D, Nakada-Tsukui K, Okada M, Nozaki T (2006). Two cysteine protease inhibitors, EhICP1 and 2, localized in distinct compartments, negatively regulate secretion in *Entamoeba histolytica*. FEBS Lett.

[b39] Scory S, Caffrey CR, Stierhof YD, Ruppel A, Steverding D (1999). *Trypanosoma brucei*: killing of bloodstream forms *in vitro* and *in vivo* by the cysteine proteinase inhibitor Z-Phe-Ala-CHN__2__. Exp Parasitol.

[b40] Seyfang A, Mecke D, Duszenko M (1990). Degradation, recycling, and shedding of *Trypanosoma brucei* variant surface glycoprotein. J Protozool.

[b41] Smith BO, Picken NC, Westrop GD, Bromek K, Mottram JC, Coombs GH (2006). The structure of *Leishmania mexicana* ICP provides evidence for convergent evolution of cysteine peptidase inhibitors. J Biol Chem.

[b42] Stempin C, Giordanengo L, Gea S, Cerbán F (2002). Alternative activation and increase of *Trypanosoma cruzi* survival in murine macrophages stimulated by cruzipain, a parasite antigen. J Leukocyte Biol.

[b43] Szoor B, Wilson J, McElhinney H, Tabernero L, Matthews KR (2006). Protein tyrosine phosphatase TbPTP1: a molecular switch controlling life cycle differentiation in trypanosomes. J Cell Biol.

[b44] Tabel H, Kaushik RS, Uzonna J (1999). Experimental African trypanosomiasis: differences in cytokine and nitric oxide production by macrophages from resistant and susceptible mice. Pathobiology.

[b45] Tasker M, Wilson J, Sarkar M, Hendriks E, Matthews K (2000). A novel selection regime for differentiation defects demonstrates an essential role for the stumpy form in the life cycle of the African trypanosome. Mol Biol Cell.

[b46] Tomás AM, Kelly JM (1996). Stage-regulated expression of cruzipain, the major cysteine protease of *Trypanosoma cruzi* is independent of the level of RNA. Mol Biochem Parasitol.

[b47] Troeberg L, Morty RE, Pike RN, Lonsdale-Eccles JD, Palmer JT, McKerrow JH, Coetzer TH (1999). Cysteine proteinase inhibitors kill cultured bloodstream forms of *Trypanosoma Brucei Brucei*. Exp Parasitol.

[b48] Troeberg L, Chen X, Flaherty TM, Morty RE, Cheng M, Hua H (2000). Chalcone, acyl hydrazide, and related amides kill cultured *Trypanosoma Brucei Brucei*. Mol Med.

[b49] Turner CMR, Aslam N, Dye C (1995). Replication, differentiation, growth and the virulence of *Trypanosoma brucei* infections. Parasitology.

[b50] Van Deursen FJ, Shahi SK, Turner CMR, Hartmann C, Guerra-Giraldez C, Matthews KR, Clayton CE (2001). Characterisation of the growth and differentiation *in vivo* and *in vitro*-of bloodstream-form *Trypanosoma brucei* strain TREU 927. Mol Biochem Parasitol.

[b51] Vicik R, Hoerr V, Glaser M, Schultheis M, Hansell E, McKerrow JH (2006). Aziridine-2,3-dicarboxylate inhibitors targeting the major cysteine protease of *Trypanosoma brucei* as lead trypanocidal agents. Bioorg Med Chem Lett.

[b52] Ziegelbauer K, Quinten M, Schwarz H, Pearson TW, Overath P (1990). Synchronous differentiation of *Trypanosoma brucei* from bloodstream to procyclic forms *in vitro*. Eur J Biochem.

[b53] Ziegelbauer K, Stahl B, Karas M, Stierhof Y-D, Overath P (1993). Proteolytic release of cell surface proteins during differentiation of *Trypanosoma brucei*. Biochemistry.

